# Neural Mechanisms of Subliminal Mentor-Student Relationship Stimuli Processing: An ERP Study

**DOI:** 10.3390/ijerph19052760

**Published:** 2022-02-27

**Authors:** Yang Wu, Na Luo, Yan Zhang

**Affiliations:** 1 School of Marxism, Huazhong University of Science and Technology, Wuhan 430074, China; wuy3814@hust.edu.cn; 2 Research Center for Social Psychology, Central China Normal University, Wuhan 430079, China; 3 School of Educational Science, Huazhong University of Science and Technology, Wuhan 430074, China; 2016210094@hust.edu.cn

**Keywords:** mentor-student relationships, ERPs, P2, N2, name processing

## Abstract

In educational contexts, mentorship roles often complicate the mentor-student relationship because mentors act not only as the closest academic ally of graduate students but also their program supervisors who can affect their timely graduation. This study examines how graduate students react to their mentors’ names when subliminally presented. A total of 63 graduate students (31 male; Mean Age = 23.450) were asked to perform an irrelevant color judgment task of valenced words (positive vs. negative relationship words) after a subliminal presentation of three different types of names (i.e., mentors, authorities, and friends). Results show that mentor and friend names elicit a greater P2 peak than authority names, whereas mentor names evoke a reduced N2 and P3 amplitude than friend and authority names. In addition, participants with a history of abusive supervision tend to have an overall decline in P2 amplitude. These event-related potential (ERP) findings suggest that mentors are perceived by students as familiar while attention-inducing figures.

## 1. Introduction

Mentorship plays a critical role in the personal and academic development of graduate students [1,2,3,4]. During the mentoring process, a healthy and productive mentor-student relationship can greatly enhance the academic skills of graduate students, facilitate their career development, and increase their general satisfaction with the graduate program [5,6,7,8]. 

Although the main goal of the mentorship system is to provide academic training for students, the mentor-student relationship still falls within the broad category of interpersonal relationships and is thus subject to “the full spectrum of human relationship disturbances” [6]. A typical mentor-student relationship often involves a mixture of different types of relationships in addition to academic advising relations, such as cooperation, friendship, admiration, supervision and, in certain circumstances, employment relationships [9,10]. Conflicts are often inevitable among these multiple co-existing aspects of a mentor-student relationship [6,9]. On the one hand, despite guiding the academic careers of students, mentors often form a partnership with these students in their research activities, nurturing them with expert knowledge and opportunities, thereby resulting in a deep intellectual integration [7,11] and a continuation of research topics, e.g., [12]. In this case, a student is usually close to his/her mentor throughout his/her academic life. On the other hand, the mentor-student relationship is inherently unequal; mentors are almost always in a position of authority relative to students [6,9]; and among all sorts of social roles with authority and status, the mentor role belongs to a subset of such roles that were equipped with genuine powers. For example, beyond the authority status, mentors usually hold the actual power of evaluating the academic performance of students, allocating research resources in the laboratory, and deciding whether students are eligible for graduation. In these cases, students, as mentees, are relatively powerless. A large body of research on power has shown that interpersonal power differentials can lead to asymmetric social distance, which makes high-power individuals feel distant [13] and triggers inhibitory or avoidance behavior, such as vigilance, fear, passivity, submission, and compliance among relatively low-power individuals [14,15,16,17]. High-power individuals also tend to engage in unethical behaviors, including workplace abuse and sexual harassment [18,19,20]. While conflicting with each other, both aspects are necessary and are internal to the mentorship relationship. Lacking power and authority, mentors would lose the privilege to evaluate students and to set academic requirements. This would turn mentorship into a loose link, as mentors can no longer make sure that mentees are capable and responsible academic partners. Lacking the partnership aspect, a mentorship relationship would easily degenerate into an exploitative or a detached one. In either case, mentorship, in the literal meaning of the term, would disintegrate and morph into something completely different. All of this makes the mentor-student relationship different from the usual hierarchical relationship or purely academic comradeships. Understanding the nuances could benefit mentors and students, policymakers, and school administrators.

Taken together, given the two co-existing aspects of mentorship and a growing literature on mentorship’s influence on students’ careers and psychological outcomes, an interesting question is how deeply the mentorship relationship could affect the graduate student. Is it possible that being in a mentorship could affect students’ early attentional processing? If so, can the experience of abusive supervision and mentorship satisfaction accentuate or attenuate such influence? This study aims to examine how graduate students react to the subliminally presented names of their mentors and the possible influence of abusive supervision and mentorship satisfaction. Specifically, event-related potential techniques were employed to explore whether key ERP components related to familiarity/closeness or threatening/attention-capturing features could differentiate between the two aspects of the mentor. A strength of the ERP technique is its non-requirement for participants to explicitly evaluate the mentoring quality of their mentors, thereby reducing the possible effects of social desirability.

The psychological presence of the social world around us could strongly affect our emotions and task performances, shift our perception of information, and by doing so, initiate a chain of reaction in our cognitive processes that often result in societal outcomes [21,22,23]. Various social cues served as reminders of the social world, and one of the most potent cues is the name. Repeated co-occurrence of names and certain emotions or motivations in the social world constitutes a conditioning process [24,25], through which the names would become valenced over time. As a result, the appearance of the names could affect one’s cognitive processes. For example, a study using dot-probe task found that an attentional bias exists over the name of the person whom one envies [26]. Several studies also revealed that even a subliminally presented name of a role model can engage and enhance participants’ social comparison tendencies [27,28]. Similarly, during the mentorship process graduate students may also form an automatic mental association between mentor names and specific attentional or other emotional responses. In that case, the presentation of mentor names would be able to generate a rapid attention allocation pattern depending on the mentorship experience.

A growing body of research using ERP and brain imaging techniques has examined individuals’ neural processing of names [29,30,31,32,33,34]. Some of these studies have examined the self-referent effect of the P300 component [29,35] and the cultural differences in name processing [34]. Generally, people direct more attentional resources to the names of familiar or important others as to their own names than to the names of unknown persons [32,35,36], thereby engendering differences in their ERP components, such as P2 and P3. For example, in a study involving different types of names, P200 amplitudes were greater for self- and other- names than names of unknown persons [32]. However, to date, the line of research on the neural processing of names has focused on the names of self, family members, friends, and strangers instead of the names of people with special roles, thereby restricting the generalizability of the aforementioned results. Besides, the previous research mostly present names supraliminally, and did not examine the effect of subliminally presented names. To fill such gap, this study examines the neural processing of mentor names by using ERP, especially the early P2 and N2 components that are typically modulated by early cognition and attention processes.

ERP studies show that the early positivity P2 component usually peaks at around 200 ms after the stimulus onset and is associated with the attention allocation process during the early stages of cognitive processing [37]. Therefore, this component is often used to index the general depth of encoding of a stimulus [38]. Previous studies show that the P2, and late P1 components are modulated by the rarity [39], spatial proximity [40], deviance e.g., violation of common knowledge [37]; affective valence [41], and threateningness [42] of the stimulus. Moral violation and moral dilemmas have been shown to elicit an enlarged P2 wave [37,43,44]. A common theme running through this cluster of stimulus features associated with P2 is that they are all linked, in varying degrees, to a heightened necessity for attention allocation. According to the hybrid model of social information processing [45], objects in visual inputs are engaged in a biased competition for attention [46], and these features attract attention probably because they are either physically salient or especially relevant regarding personal goals or contexts [46,47,48] that demands an immediate response.

Similar to P2, N2 is an early sensory component that usually peaks from 180 ms to 325 ms. Researchers generally believe that N2 reflects stimulus novelty, expectation violation, or cognitive control [44,49,50,51,52]. The link between the N2 component and unfamiliar, novel stimulus was first documented in early ERP studies [49,53]. The N2 ERP is evoked in response to novel stimuli presented in auditory [53], visual [51,54], and facial recognition tasks [55]. A recent study shows that the sensation-seeking trait (i.e., a personality tendency to seek novel and different experiences) also modulates N2 peak amplitudes, that is, when presented with a novel stimulus, high sensation seekers demonstrate a high N2 amplitude [56].

Previous studies also found that a frontocentral N2 component is related to cognitive control, especially in various forms of cognitive conflicts [50]. In a variety of paradigms pertaining to cognitive control or stimulus-response conflicts, including spatial Simon tasks [57], Stroop tasks [58], Eriksen Flanker tasks [59], and approach-inhibition tasks [52], larger N2 components are shown to accompany conflict processing. This N2 effect has also been observed in the conflict processing of social stimuli. For example, a recent study shows that those behaviors violating moral norms elicit a larger N2 [44].

In the present study, to examine the rapid attentional pattern associated with the mentor, we select three types of name primes, namely, the names of the mentor, a friend (high familiarity), and an authority (high status), with an aim to tease apart the two aspects of mentorship by examining the differences in P2 and N2 waves following these primes. Based on previous ERP literature, we predict a significant modulation of P2 and N2 components as a function of the name primes. As mentors usually have higher power, while “the powerless attend to the powerful who controls their outcome” [60], it is possible that the mentor name could be an attention-capturing cue that marks the mentor’s social presence for students and could elicit a more pronounced P2 wave. On the other hand, as mentors are usually close to graduate students through constant academic interaction, the mentor name prime should be distinguishable from names that are with authority but not close to the students. Had this been the case, the novelty-related N2 should be attenuated following the mentor name prime but not the authority name prime.

Apart from P2 and N2, we also include P3 in our analyses because of its link with the familiarity of names as revealed in the name processing literature [30,32,35,36,49]. As a major positivity peak apparent at 300 ms after the stimulus onset, P3 was found to be modulated by stimulus intensity, significance, probability (novelty), and infrequency [30,36,51,61,62], though the underlying mechanisms and the structure of its various subcomponents (e.g., the frontal P3a and posterior P3b) were often in debate [61,63]. More pertinent to the present study, research on name processing found that name familiarity or novelty could modulate P3 amplitude [32,36]. Besides, several studies also found that subliminal and supraliminal priming could modulate P3 components [64,65,66,67]. Therefore, we expect that P3 amplitude could also be affected by the subliminally presented names of different levels of familiarity/novelty, as N2.

Unlike past research on name processing, the present study adopted a subliminal priming paradigm, e.g., [68], and after subliminally presenting the name primes, participants were instructed to finish an irrelevant Stroop task, where positive and negative relationship words were presented with either red or green colors. The aim is to examine whether the subliminal priming could affect the early cognitive process in the relatively easy task, as measured through ERP components of P2, N2, and P3. For exploratory purposes, we also asked participants to finish questionnaires on mentor abuse and mentor satisfaction to check whether the experience of being abused during supervision and self-reported mentor satisfaction are related to these effects.

## 2. Methods

### 2.1. Participants

Sample size was determined using Gpower 3.1.3 [69]. With alpha error probability set at 0.05, power set at 0.90, and a conservative estimate of effect size set at 0.20 (Cohen’s f), the sample size was calculated to be 55. We recruited 20% more participants to offset potential dropouts. Sixty-six right-handed participants were recruited at a research university from central China via classroom invitation and social media. Initial screenings of the EEG data excluded 3 participants due to excessive artifacts, leaving a valid sample of 63 participants (31 males) for the main ERP study. Ages of the participants ranged from 21–33 years (*M* = 23.450, *SD* = 1.912). In addition, seven participants did not finish the questionnaires on mentor abuse and mentor satisfaction (reasons include refusal and incompleteness in responding). Their data were retained for ERP study but were excluded from the correlational study. Due to the sensitive nature of some of the questionnaire items, key personal identifying information, such as names, students’ IDs, was not collected in the study to protect the students’ privacy. Moreover, participants’ questionnaire responses and ERP data were kept confidential and were stored and used in a de-identified form. Before participation, all participants were informed about the goal, procedures, and privacy protection of the study and signed the informed consent.

### 2.2. Materials

A raw list of 100 words related to mentor-student relationship was collected by searching dictionaries. Twenty-one graduate students of psychology were invited to rate the list of words on arousal (1 = *very relaxing*, 5 = *medium*, 9 = *very arousing*) and valence (1 = *very negative*, 5 = *medium*, 9 = *very positive*), along a 9-point Likert scale. To derive two lists of words differing on valence while equivalent on arousal level, the words were selected and ordered into two sets according to the ratings before testing inter-set differences on both dimensions; words were further adjusted until the criteria were met. The whole iterative process was repeated several times to achieve equivalence on arousal level while being significantly different on valence. The final word lists consisted of 40 words (20 positive words and 20 negative words). The positive words (6.95 ± 1.17) differed significantly from the negative words (3.03 ± 0.86) on valence, *F*(1, 20) = 101.237, *p* < 0.001. The arousal level between the two sets did not differ significantly (5.75 ± 1.18 vs. 5.22 ± 1.77; *p* > 0.08), nor did the visual complexity (character stroke numbers: 16.35 ± 4.63 vs. 16.00 ± 3.97; *p* = 0.80). The stimulus word frequency was retrieved through the “Chinese 2019” corpus in Google Ngram using the R package “ngramr” [70]. The word frequency of each word through 1900–2018 was averaged to form an overall word frequency index. A comparison between positive and negative words showed no significant difference in word frequency, *t*(38) = 0.59, *p* = 0.56. All words were presented as pictures (size: 212 × 115 pixels). Examples of positive words are trust, frankness, supportiveness; negative words are pressure, exploitation, estrangement. All words were in Chinese.

Prior to the experiment, participants were asked to provide the full names of their mentors and one of their friends. These two names, together with the name of a deceased national leader in history (Mao Zedong) used for Authority condition, constituted the name primes in the experiment. An additional 78 participants (*M*_age_ = 19.61, *SD* = 0.84) were recruited to rate Mao Zedong and other 14 filler items on perceived authoritativeness along a 7-point Likert scale. Results showed that the name of Mao Zedong was perceived as highly authoritative (*M* = 6.68, *SD* = 0.69), which is significantly higher than the mid-point, *t*(77) = 34.14, *p* < 0.001.

### 2.3. Psychometric Assessment

#### 2.3.1. Mentor’s Abusive Supervision

The Chinese version [71] of the Abusive Supervision scale [72] was adopted to measure the extent to which participants experienced abusive supervision from their mentors. The scale consisted of 15 items of abusive supervisory behaviors (e.g., “ridicule me”, “tells me my thoughts or feelings are stupid”), to which participants rated the frequency on a 7-point Likert scale (1 = *very rare* to 7 = *very often*). In the present study, Cronbach α = 0.954.

#### 2.3.2. Mentor Satisfaction

A single face-valid item was employed to measure participants’ satisfaction with their mentors, “To what extent do you feel satisfied about the guidance you received from your mentor?” Participants rated their agreement on a 7-point Likert scale (1 = *very unsatisfactory* to 7 = *very satisfactory*).

### 2.4. Experimental Procedure

Following electrode application, participants sat in front of a computer screen with a distance of about 80 cm from the eyes. The words were presented on a Dell 27-inch LCD monitor in a 24 point Songti font (height of 2 cm and width of 3 cm).

**Figure 1:**
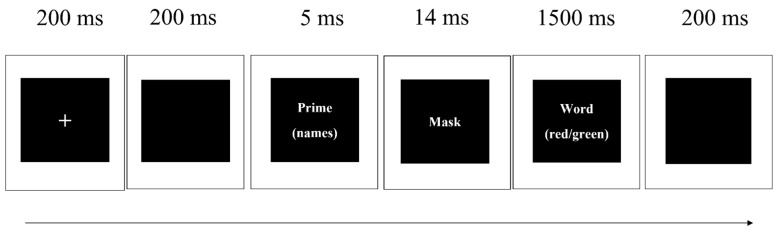
Procedure of experiment.

Experimental materials were presented using E-Prime 3.0. Each experimental trial (Figure 1) started with a white 200-ms fixation “+” and a 200-ms blank screen. Subliminal name primes appeared for 5 ms, followed by a 14-ms mask. The mask was a string of letters (“AABB”) and covered the space of the prime stimuli. After the mask, the word stimuli appeared for a maximum of 1500 ms, during which the participants were asked to judge the word color as soon by pressing 1 for red words and 2 for green words, irrespective of the word meaning. The word stimuli disappeared once the participant responded. It was then followed by another 200-ms blank screen. The order for all conditions and stimuli word was randomized.

The experiment consisted of 2 blocks, with a total of 240 trials for the 6 stimulus pairings (each has 40 trials) and a short rest between the blocks. Therefore, for each participant, the names for the mentor, the friend, and the authority figure each appear 80 times, respectively. Before the formal experiment, there are 8 practice trials to ensure that participants understand the experimental procedures. The whole experiment took about 10 min. After the experiment, each participant was thoroughly probed for suspicion or awareness of the subliminal primes. None of the participants reported seeing the prime words.

### 2.5. ERP Data Recording

The electroencephalograph (EEG) voltages from 64 scalp sites were recorded by Brain Vision Recorder software (Version 1.10, Brain Products GmbH, Munich, Germany) and all sites were referenced to the left and right mastoids (average mastoid reference). The ground electrode was on the medial frontal aspect. Electrode impedances were kept below 5 kΩ. Sampling rate was 500 Hz and bandpass filter was set to 0.05–80 Hz.

Offline data analyses were conducted using Brain Vision Analyzer (Version 2.1, Brain Products GmbH, Munich, Germany). To derive the ERP components, the continuous EEG was segmented to include only the (−200, 1000 ms) epochs time-locked to the word stimulus onset, and baseline correction was applied over the 200 ms preceding the word onset. An artefact rejection excluded all segments with amplitudes exceeding ±80 µv, bursts of electromyographic (EMG) activity, eye movements or trials with incorrect behavioral responses. Overall, this process rejected 9.49% of trials across participants and across conditions. The average percentages of trials of each condition that survived the artifact rejection are 0.92 ± 0.14 (authority-positive), 0.91 ± 0.16 (authority-negative), 0.90 ± 0.14 (mentor-positive), 0.90 ± 0.16 (mentor-negative), 0.90 ± 0.16 (friend-positive), 0.91 ± 0.14 (friend-negative)

We determined the timing, topography and electrodes of ERP components based on previous research, e.g., for P2 electrodes in priming paradigm [73,74]; combined with visual inspection of empirical topographical maps and grand-averaged waveform collapsed across all participants and conditions. Due to the closeness of the components in time and the individual differences in latency across the relatively large sample of participants, we set slight overlaps in time windows of adjoining components [75] to ensure that across all the participants each component could actually peak within the time window. A close visual inspection of waveforms of each participant ensured that no component mix-up occurred. Following these steps, the ERP time window and electrodes chosen include P2 (100–200 ms) from central (C3, C4, Cz) and centro-parietal (CP3, CP4, CPz) regions, N2 (180–280 ms) from frontal (F3, F4, Fz), central (C3, C4, Cz) and parietal (P3, P4, Pz) regions and P3 (250–350 ms) from central (C3, C4, Cz), central-parietal (CP3, CP4, CPz), frontal (F3, F4, Fz) and parietal (P3, P4, Pz) regions.

### 2.6. Statistical Analysis

We used repeated measure analyses of variance (ANOVAs) to test the effect of mentor name prime on ERP peak amplitudes, and adjusted *p* value according to Greenhouse–Geisser formula. For significant main effects or interaction effects, post-hoc and simple effect testing was conducted using Bonferroni correction.

The judgement task accuracy and reaction time were separately subjected to 3 (name prime: mentor/authority/friend) × 2 (valence: positive/negative) repeated-measure ANOVA. The P2 amplitude was subjected to a 3 (name prime: mentor/authority/friend) × 2 (valence: positive/negative) × 2 (brain regions: central/centro-parietal) three-way repeated measures ANOVA. The N2 amplitude was subjected to a 3 (name prime: mentor/authority/friend) × 2 (valence: positive/negative) × 3 (brain regions: frontal/central/parietal) three-way repeated measures ANOVA. The P3 amplitude was subjected to a 3 (name prime: mentor/authority/friend) × 2 (valence: positive/negative) × 4 (brain regions: frontal/central/parietal/centro-parietal) three-way repeated measures ANOVA.

Structural equation modelling [76] and multilevel regression was used to test the effects of mentor abusive supervision and mentor satisfaction. Specifically, we conducted a series of multilevel regression using lme4 package in R [77] to test the potential moderating effects of abusive supervision and mentor satisfaction on ERP amplitudes. In each model, the ERP amplitudes of N2, P2, and P3 acted as the criterion, respectively. Level-1 predictors were effect-coded categorical variables for experimental conditions, including brain regions, name prime, and valence. Two level-2 predictors, abusive supervision, and mentor satisfaction, served as moderators respectively.

## 3. Results

### 3.1. Behaviors

None of the main effects and interaction effects reached significance. The mean RT across the conditions ranged from 481.62 (*SD* = 63.22) to 487.66 (*SD* = 60.93) ms, while the mean accuracy rates ranged from 0.97 (*SD* = 0.3) to 0.98 (*SD* = 0.3).

### 3.2. ERPs

#### 3.2.1. P2

**Figure 2:**
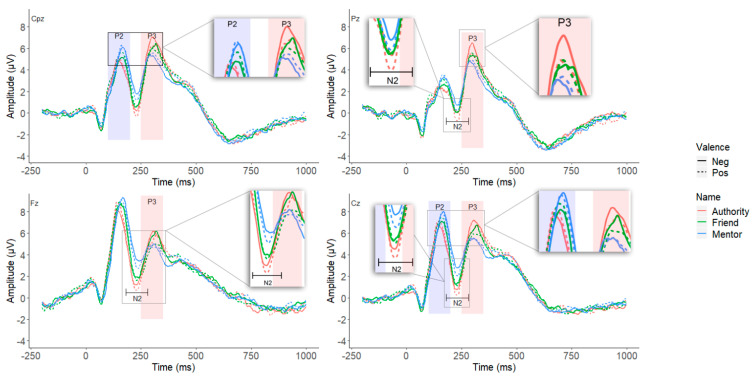
Grand average ERPs of the P2, N2 and P3 components at the indicated electrode sites.

**Figure 3:**
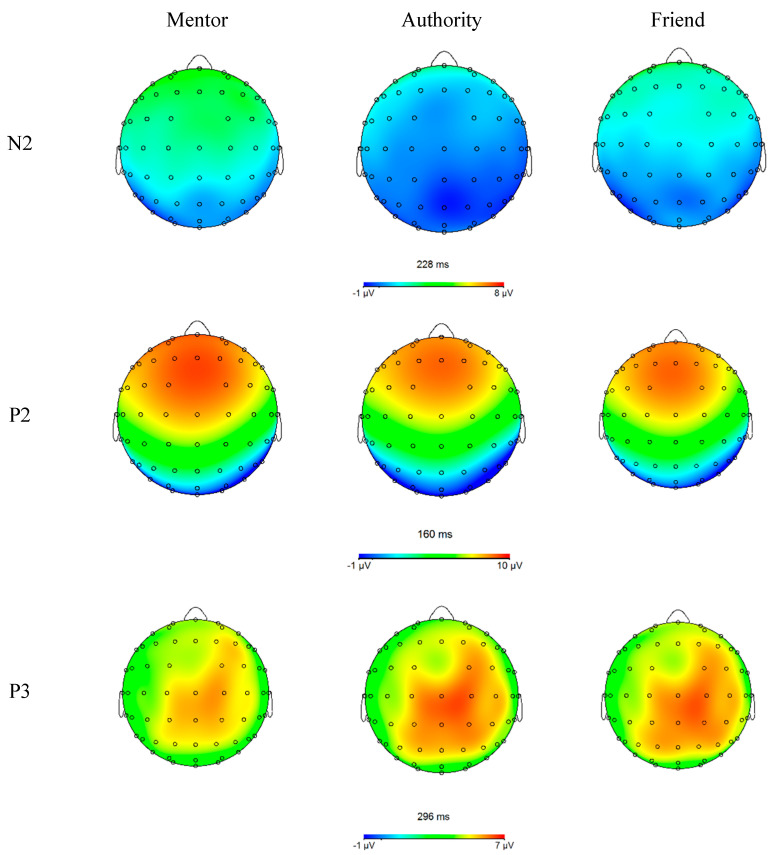
Topographical maps of the voltage amplitudes of N2 (Fz), P2 (Cz) and P3 (Pz) for each name prime condition.

As shown in Figure 2 and Figure 3, the main effect of name prime was significant, *F*(2,124) = 3.682, *p* = 0.030, η_p_^2^ = 0.056. Mentor name prime elicited larger amplitudes (*M* = 8.167 μV, *SE* = 0.383) than authority name condition (*M* = 7.641 μV, *SE* = 0.412, *p* = 0.048) but not the friend name condition (*M* = 7.979 μV, *SE* = 0.406, *p* = 0.720); and no significant differences existed between authority and friend name conditions (*p* = 0.186). A planned contrast showed that when treating mentor and friend as a whole, it elicited overall larger P2 amplitudes than authority, *F*(1,62) = 6.538, *p* = 0.013, η_p_^2^ = 0.095. The main effect of brain regions was also significant, *F*(2,124) = 132.134, *p* < 0.001, η_p_^2^ = 0.681, as P2 amplitudes were greater in central (*M* = 8.786 μV, *SE* = 0.391) than centro-parietal area (*M* = 7.072 μV, *SE* = 0.392).The three-way interaction was marginally significant, *F*(2,124) = 2.639, *p* = 0.077, η_p_^2^ = 0.041. Further analysis revealed that the simple interaction of brain area × valence was significant in authority name condition, *F*(1,62) = 4.580, *p* = 0.036, η_p_^2^ = 0.069, but not significant in either mentor name or friend name conditions. Decomposing this significant simple interaction in the authority name condition, the results showed that for both valence conditions, larger P2 amplitudes were observed in central area (*M*_pos_ = 8.586 μV, *SE* = 0.427; *M*_neg_ = 8.400 μV, *SE* = 0.447) than centro-parietal area (*M*_pos_ = 6.776 μV, *SE* = 0.431; *M*_neg_ = 6.804 μV, *SE* = 0.431), yet the magnitude of difference was greater in positive words condition (Mean Difference = 1.810 vs. 1.596; *p*s < 0.001). All the remaining main effects and two-way interactions were not significant. 

#### 3.2.2. N2

The main effect of name prime was significant, *F*(2,124) = 24.637, *p* < 0.001, η_p_^2^ = 0.284. Authority (*M* = −0.575 μV, *SE* = 0.380) and friend name primes (*M* = −0.072 μV, *SE* = 0.355) conditions all showed more pronounced N2 amplitude than mentor name prime (*M* = 1.010 μV, *SE* = 0.337; *p*s < 0.001), with no significant differences between authority and friend (*p* = 0.114). The main effect for brain regions was also significant, *F*(2,124) = 8.708, *p* = 0.003, η_p_^2^ = 0.123. The parietal N2 (*M* = −0.378 μV, *SE* = 0.302) was greater than central (*M* = 0.190 μV, *SE* = 0.353, *p* = 0.002) and frontal regions (*M* = 0.552 μV, *SE* = 0.407, *p* = 0.010), with the latter two having no significant differences (*p* = 0.167).

**Figure 4:**
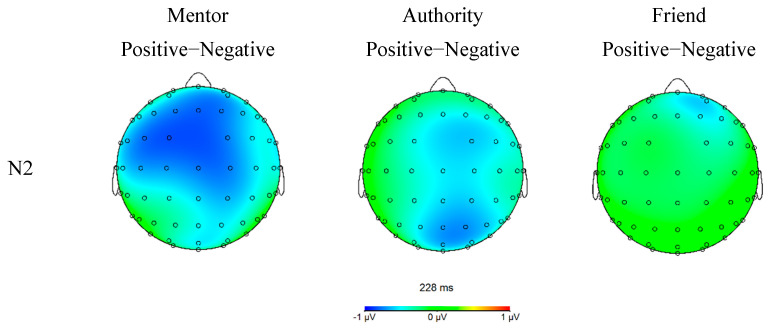
Topographical map of difference waves of N2 interaction.

The two-way interaction of name prime and valence was significant, *F*(2,124) = 3.771, *p* = 0.026, η_p_^2^ = 0.057. As shown in Figure 4, simple effect analysis showed that the positive words (*M* = 0.698 μV, *SE* = 0.350) condition showed greater N2 amplitude than the negative words condition (*M* = 1.323 μV, *SE* = 0.361) only when primed by the mentor name (*p* = 0.007), but not by authority (*M* = −0.715 vs. −0.413 μV, *SE* = 0.381 vs. 0.405; *p* = 0.165) or friend names (*M* = 0.050 vs. −0.194 μV, *SE* = 0.366 vs. 0.387; *p* = 0.335). A marginal significant two-way interaction was found between brain regions and name prime, *F*(2,124) = 2.643, *p* = 0.066, η_p_^2^ = 0.041. The simple main effect of name prime was slightly weaker in parietal than frontal and central regions. The remaining main effect and interaction effects were not significant.

#### 3.2.3. P3

The main effect of name prime was significant, *F*(2,124) = 14.118, *p* < 0.001, η_p_^2^ = 0.185. Authority (*M* = 7.864 μV, *SE* = 0.390) and friend name primes (*M* = 7.431 μV, *SE* = 0.389) conditions all showed more pronounced P3 amplitude than mentor name prime (*M* = 6.857 μV, *SE* = 0.370; *p*s < 0.020), with a marginal significant difference between authority and friend (*p* = 0.055).

The main effect for brain regions was also significant, *F*(3,186) = 5.594, *p* = 0.012, η_p_^2^ = 0.083. The parietal P3 (*M* = 6.854 μV, *SE* = 0.385) was slightly weaker than central (*M* = 7.763 μV, *SE* = 0.392, *p* = 0.002) and centro-parietal regions (*M* = 7.484 μV, *SE* = 0.386, *p* < 0.002), with the latter two having no significant differences (*p* = 0.121). The difference between parietal region and frontal region (*M* = 7.436 μV, *SE* = 0.408) did not reach significance (*p* = 0.556).

In addition, the main effect of valence was marginally significant, *F*(1,62) = 3.406, *p* < 0.070, η_p_^2^ = 0.052, such that negative words (*M* = 7.514 μV, *SE* = 0.380) showed a tendency to elicit larger P3 ERP than positive words (*M* = 7.254 μV, *SE* = 0.368). All other two-way and three-way interactions were not significant.

### 3.3. Correlation between ERPs and Subjective Ratings

To examine the influence of abusive supervision (*M* = 1.352, *SD* = 0.618) and mentor satisfaction (*M* = 4.714, *SD* = 1.331), the mean score of both variables were used to calculate the correlation with P2 and N2 ERP amplitudes. Descriptive statistics showed that the general level of abusive supervision was low, as the mean score ranged only from 1 to 3.467 on a 7-point Likert scale. The correlation between abusive supervision and mentor satisfaction was significant (*r* = −0.550, *p* = 0.001).

**Figure 5:**
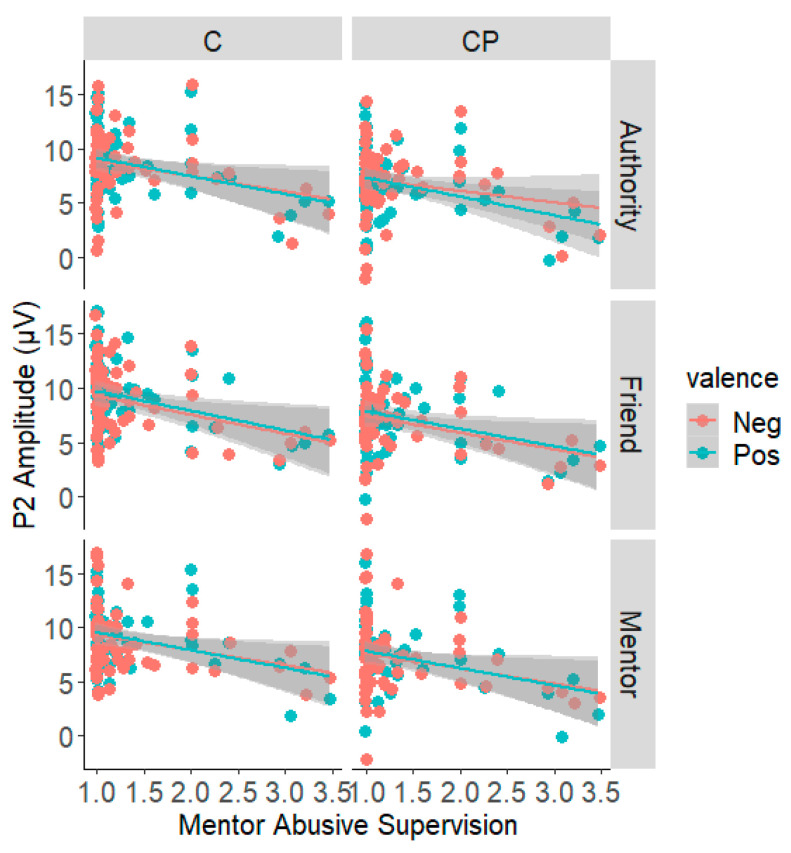
Association between abusive supervision and P2 amplitude.

As shown in Table 1 and Figure 5, the experiences of abusive supervision exhibited an overall negative correlation with P2 amplitudes in nearly all conditions. A test on the dependent correlational pattern based on structural equation modelling [76] confirmed that this pattern of negative correlation does not significantly vary across experimental conditions, χ^2^(11) = 1.16, *p* > 0.999. Besides, no significant association was found between mentor’s abusive supervision and N2 component; self-report mentor satisfaction also showed no correlation with both P2 and N2 ERPs.

We further examined the potential interaction among the experimental conditions and the abusive supervision or mentor satisfaction by using mixed modeling. The results found that all interactions involving abusive supervision or mentor satisfaction were not significant.

## 4. Discussion

This study aims to identify the ERP components involved in the neural responses of graduate students to the names of their mentors and to examine the dual aspects of mentorship and the implicit affective evaluation of the mentors. The participants were presented with different name primes before they were asked to rate a series of affective words during which the ERP data were recorded. Results show that the mentor and friend name primes elicit larger P2 potentials than the authority name prime and that the mentor name prime induces a lower N2 and P3 amplitude than the authority and friend name primes. Moreover, the pairing of mentor name with positive words has a larger N2 effect than the pairing with negative words.

In the present study, the mentor and friend name primes both induce a greater P2 amplitude than the authority name prime, and no significant differences are observed between mentor and friend names. In previous research, an increase in P2 and late P1 potentials is often accompanied by various attention-capturing features of the stimulus, such as rarity [39], spatial proximity [40], and affective intensity [41,42,75,78]. Given the close relationship between P2 and attention allocation [73], the above finding can reflect the relative salience of mentor and friend relative to authority from the perspective of graduate students. The authority name prime used in the experiment was a deceased historical figure who is distant from the participants both temporally and spatially, and no longer has actual power to influence people. The mentor and friend, on the other hand, were living persons close and important to the participants either personally or academically and could have real influence on them. Therefore, the mentor and friend name primes are more attention-inducing in the early visual processing than the authority name prime.

For N2, the present study found that the mentor name prime induced a reduced N2 amplitude than both the friend and authority name primes, and a significant interaction is observed between these name primes and the valence of affective words such that positive words elicit a larger N2 amplitude than negative words only when primed by mentor names. The current result that N2 potential was reduced after the subliminal presentation of the mentor name (and the parallel increases in P3 amplitude) is generally consistent with previous research on N2 and P3 familiarity effects. In the related literature, N2 is shown to be associated with stimulus novelty, expectancy violation, and cognitive controls [44,49,50,51,52]. As for the novelty effect, familiar and less novel stimuli often elicit weak N2 and P3 effects [55,79] given that these components can reflect stimulus novelty or infrequency [50,61]. However, being objectively frequent or infrequent is often insufficient to affect N2 magnitude or P3 novelty as previous studies have highlighted the context dependency of such familiarity effects and suggested that stimuli “must be either highly unfamiliar and thus deviate from long term context, or deviate considerably from short term context” for a review, see [50]. As graduate students, the participants found the presentation of mentor name as less novel perhaps because the experiment took place during the semester, a time when these students have ample daily interactions with their mentors, and at a laboratory of the university, where the names of these mentors are treated as natural parts of the academic system. Conversely, the name of a friend or a deceased historical authority sharply contrasts the laboratory setting (short-term context) or the postgraduate education in which the participants are engaged (long-term context).

Furthermore, the main effect of N2 topographies from the present study also revealed an elevated posterior N2, which could be interpreted as indicating visual attention to the task requirement as well as the novel or infrequent stimulus [50]. Yet given that the novelty N2 generally has a more anterior distribution [50,56], this seems to cast doubt on the viability of the mentor-familiarity interpretation. However, the decomposition of two-way interaction showed that despite the main effect of brain regions, the name prime effects of N2 is more pronounced in frontal and central regions, supporting the leading role of anterior N2 in novelty-detection. In addition, there was ample evidence that the posterior N2 subcomponents were also sensitive to stimulus novelty [50,79,80], depending on the modality or task requirements. Future studies using devices with higher spatial resolution could further determine the brain regions involved in the mentor name effect.

The present study also had an unexpected finding that positive words (vs. negative words) elicit a greater N2 effect after the subliminal presentation of the mentor name. Given previous research that use N2 as an indicator of expectation violation or cognitive control, a possible interpretation of this result could be that a high cognitive incongruence exists between mentor name and positive words. Extensive literature on implicit social cognition is predicated on the basic consensus that tasks that involve incongruence in priming stimuli and target stimuli will implicate a top-down cognitive control (e.g., response inhibition), thereby reducing response accuracy and increasing reaction time [81,82,83], both of which are used as measures of implicit attitude in several implicit tasks, such as implicit association tests (IAT). For example, a longer reaction time or a higher error rate over the pairing of “Object A” and “Good” may suggest an implicit negative view of Object A. Recent studies also identify N1 and N2 ERP components as indicators of such stimuli incompatibility effect [44,52]. Therefore, the findings of this work can be understood in terms of the abovementioned literature as suggesting a more negative attitude toward mentors. Unfortunately, due to the exploratory nature of the present study, the behavioral task in the present study only required the participants to judge the color, but not the meaning, of the affective words, unlike paradigms such as IAT. The reaction time or error rates of the color judging task could scarcely reflect the possible incompatibility between name prime and the affective words, thus rendering the behavioral data nondiagnostic of the incompatibility effect. Therefore, the behavioral responses recorded in this study are not comparable with those reported in the literature. Nonetheless, the unexpected discovery of the N2 deflection at mentor-positive words pairings provided a promising avenue for future research, though we caution that this result is still preliminary and further replication is imperative. Future studies should employ classical implicit social cognition tasks (such as IATs) in tandem with ERPs to further explore the cognitive representation of mentors and to examine whether the behavioral indices of implicit affective evaluation could align with the ERP waveforms associated with the mentors.

This study also examines the possible effects of abusive supervision and mentor satisfaction on ERP waves, and results show an overall reduction in P2 amplitude across all experimental conditions for those participants with high abusive supervision experiences. Meanwhile, self-reported mentor satisfaction does not show any relationship with all ERP components. This finding showed, for the first time, the abusive supervision by graduate mentors is associated with the neural activities of mentees in their early perceptual processes. Although the cross-sectional design of the present study precluded causal inferences, this finding still points to many possibilities. It could be that mentor abuse lowered P2 amplitude directly or indirectly through other psychological conditions (e.g., depression), or certain personality characteristics whose neural correlates include a relatively lower P2 amplitude may predispose students to be more easily or heavily affected by mentor abuse. Relatedly, ERP studies on post-traumatic stress disorders (PTSD) may shed some light on these findings. Several studies show that trauma-related stimuli can increase P200 amplitude among PTSD patients [84,85], while other studies report reductions in P200 amplitudes across different types of stimuli, thereby suggesting an undifferentiated attenuation of P200 magnitude brought about by trauma [86]. In the present study, the overall reduction in P2 amplitudes among students with abusive supervision experiences is consistent with the latter finding but is at odds with the former. According to a recent review, the mixed results on PTSD can be ascribed to the different paradigms and stimuli used in each study [87], which may have evoked distinct neural mechanisms. This could also be the case in the name processing of mentors in the present study if the causal relationship could be established. Therefore, future studies should replicate the present finding using different paradigms and stimuli and examine the causal relationship between P200 and abusive experiences by adopting longitudinal design.

Overall, the present results on the modulation of P2, P3 and N2 by subliminal name primes showed that the impact of mentorship could reach deep into one’s early attentional processing, suggesting that mentors may have a high social presence for graduate students. According to social presence theory [88], two critical components of being socially present are intimacy and immediacy. The intimacy refers to the perceived feeling of connectedness with an interactant, while immediacy refers to a feeling that despite being physically absent, the interactant is still “here”, seemingly able to judge, appraise and affect us. During the length of the mentoring process, mentors could not be always physically present, yet the attentional patterns in ERP components suggests that mentors were socially present on the two aspects, such that a subliminal presentation of their names can modulate ERP waveforms of the mentees. Moreover, from this perspective, mentorship involves more than power dynamics. In a pure power dynamic, at the receiving end of active power exercises, the powerless may perceive the powerholder as omnipresent, e.g., “capillary form of power” [89]; but aversive and intrusive-a case of high immediacy but low intimacy. Mentorship, on the other hand, requires a nurturing aspect for it to be functional. Future research could further examine the neural correlates of the various other aspects of mentorship in different settings.

The present finding may also have implications for understanding mentorship-like human relations in other fields. Beyond academia, the mentoring relationship is common in multiple area, such as medical professional training [90], apprenticeship in vocational education, e.g., [91], and mentoring programs in many corporations. Despite the many benefits of mentorship, most studies also identified the imbalance of power as a major threat to its effectiveness [90,92]. The results from present ERP study point to the influence of mentorship on early attentional processing, further demonstrating the huge impact that mentors may have on mentees. Thus, it is important for mentors to be more mindful as to their interaction with mentees.

### Limitations and Future Research

First, our participants were mostly students pursuing master’s degrees, with PhD students accounting for only a small proportion of our sample. The relationship between mentors and PhD students tends to be more closely knitted and more emotionally fraught than that between mentors and master students because of the higher academic ambition of the former, which strengthens the scholarly interdependence between the two. Achieving a more balanced sampling of both master and PhD students and investigating the possible differences between these students in terms of mentor-student relationship may be considered objectives for future research. Second, the aims, organizations, and role of mentors in each graduate program greatly vary across disciplines or across laboratories within the same discipline. Identifying typical modes of mentorship across disciplines and examining their influence on mentor-student relationship and the neural correlates are fruitful directions for future research. Besides, it is also possible that mentors in the workplace may have higher power than academic mentors, yet at the same time mentees in the workplace may have higher independence than graduate students. It is incumbent upon future research, therefore, to compare the mentorship in different fields. Lastly, the present study explored the association of abusive supervision, mentor satisfaction, and students’ ERP waveforms. It is important to replicate and extend the present finding in a future study, using more powerful designs and explicit measures. In addition, future studies may also explore the neural markers possibly associated with other outcome criteria for graduate programs, such as graduate students’ academic productivity, general satisfaction with the program, and the rate of graduation. Future studies may also employ devices with higher spatial resolution, such as fMRI, to better understand the neuroanatomical localization of the mentorship name processing. Adoption of impactful laboratory manipulation paradigms, such as Best Alternative to a Negotiated Agreement BATNA [93], could also help manipulate power/status effects more rigorously in future studies.

## 5. Conclusions

For graduate students, mentor and friend names elicit a stronger P2 peak than authority name, whereas mentor name evokes a reduced N2 and P3 amplitude compared to friend and authority names. These event-related potential findings suggest that mentors are perceived by students as familiar while attention-inducing figures. In addition, preliminary results from the present study suggest that participants with a history of abusive supervision tend to have an overall decline in P2 amplitude.

## Figures and Tables

**Table 1:** Association between P2 and students’ evaluation of mentorship.

Brain Region	Prime	Valence of Target Word	Abusive Supervision	Mentor Satisfaction
C	Mentor	Positive	−0.333 *	0.125
C	Mentor	Negative	−0.280 *	0.105
CP	Mentor	Positive	−0.299 *	0.141
CP	Mentor	Negative	−0.251 †	0.095
C	Authority	Positive	−0.327 *	0.107
C	Authority	Negative	−0.270 *	0.014
CP	Authority	Positive	−0.324 *	0.169
CP	Authority	Negative	−0.205	0.059
C	Friend	Positive	−0.334 *	0.110
C	Friend	Negative	−0.321 *	0.167
CP	Friend	Positive	−0.292 *	0.166
CP	Friend	Negative	−0.289 *	0.170

*Note*. * *p* < 0.05, † *p* < 0.1.

## Data Availability

Data will be stored in a publicly accessible repository and will be available upon publication from the osf.io database (osf.io/saj3p).
